# Carriage and Subtypes of Foodborne Pathogens Identified in Wild Birds Residing near Agricultural Lands in California: a Repeated Cross-Sectional Study

**DOI:** 10.1128/AEM.01678-19

**Published:** 2020-01-21

**Authors:** N. Navarro-Gonzalez, S. Wright, P. Aminabadi, A. Gwinn, T. V. Suslow, M. T. Jay-Russell

**Affiliations:** a Western Center for Food Safety, University of California—Davis, Davis, California, USA; b Sacramento City College, Sacramento, California, USA; c California Department of Fish and Wildlife, Monterey, California, USA; d Department of Plant Sciences, University of California—Davis, Davis, California, USA; University of Manchester

**Keywords:** STEC, *Salmonella*, O157:H7, produce, cattle, wildlife

## Abstract

Current California agricultural practices strive to comanage food safety and habitat conservation on farmland. However, the ecology of foodborne pathogens in wild bird populations, especially those avian species residing in proximity to fresh produce production fields, is not fully understood. In this repeated cross-sectional study, avifauna within agricultural lands in California were sampled over 1 year. Feces, oral swabs, and foot/feather swabs were cultured for zoonotic Salmonella spp., Escherichia coli O157:H7, and non-O157 Shiga toxin-producing E. coli (STEC) and characterized by serotyping and pulsed-field gel electrophoresis. Of 60 avian species sampled, 8 species (13.3%, bird groups of sparrows, icterids, geese, wrens, and kinglets) were positive for at least one of these foodborne pathogens. At the individual bird level, the detection of foodborne pathogens was infrequent in feces (*n* = 583; 0.5% *Salmonella*, 0.34% E. coli O157:H7, and 0.5% non-O157 STEC) and in feet/feathers (*n* = 401; 0.5% non-O157 STEC), and it was absent from oral swabs (*n* = 353). Several subtypes of public health importance were identified, including Salmonella enterica serotype Newport, E. coli O157:H7, and STEC serogroups O103 and O26. In late summer and autumn, the same STEC subtype was episodically found in several individuals of the same and different avian species, suggesting a common source of contamination in the environment. Sympatric free-range cattle shared subtypes of STEC O26 and O163 with wild geese. A limited rate of positive detection in wild birds provides insights into broad risk profile for contamination considerations but cannot preclude or predict risk on an individual farm.

**IMPORTANCE** The shedding dynamics of foodborne pathogens by wild birds on farmland are not well characterized. This yearlong study sampled wild birds for foodborne pathogens within agricultural lands in northern California. There was a low prevalence of *Salmonella* spp., Escherichia coli O157:H7, and non-O157 Shiga-toxin producing E. coli (prevalence, 0.34% to 0.50%) identified in bird populations in this study. However, pathogens of public health importance (such as Salmonella Newport, E. coli O157:H7, and STEC O103 and O26) were identified in fecal samples, and two birds carried STEC on their feet or feathers. Identical pathogen strains were shared episodically among birds and between wild geese and free-range cattle. This result suggests a common source of contamination in the environment and potential transmission between species. These findings can be used to assess the risk posed by bird intrusions in produce fields and enhance policy decisions toward the comanagement of food safety and farmland habitat conservation.

## Abstract (precis)

The shedding dynamics of foodborne pathogens by wild birds on farmland are not well characterized. This yearlong study sampled wild birds for foodborne pathogens within agricultural lands in northern California. There was a low prevalence of *Salmonella* spp., Escherichia coli O157:H7, and non-O157 Shiga-toxin producing E. coli (prevalence, 0.34% to 0.50%) identified in bird populations in this study. However, pathogens of public health importance (such as Salmonella Newport, E. coli O157:H7, and STEC O103 and O26) were identified in fecal samples, and two birds carried STEC on their feet or feathers. Identical pathogen strains were shared episodically among birds and between wild geese and free-range cattle. This result suggests a common source of contamination in the environment and potential transmission between species. These findings can be used to assess the risk posed by bird intrusions in produce fields and enhance policy decisions toward the comanagement of food safety and farmland habitat conservation.

## INTRODUCTION

In 2006, an Escherichia coli O157:H7 outbreak linked to prewashed packaged baby spinach was traced to the central coast of California (1), where a vast amount of the leafy greens consumed in the United States are grown (2). The outbreak strain was cultured from multiple environmental sources on agriculture land adjacent to the spinach fields, including cattle (Bos taurus) and feral pig (Sus scrofa) feces (3). The findings raised awareness regarding potential food safety risks from adjacent land use during the production of leafy greens and other fresh produce, especially those consumed raw. In response, the industry developed best food safety practices through the Leafy Green Marketing Agreement that was implemented in California and Arizona (4). Some growers currently follow comanagement approaches to balance efforts to reduce the risk from microbial contamination due to animal intrusions, while also promoting environmental stewardship of these agricultural landscapes (5). Likewise, the U.S. Food and Drug Administration’s (FDA) Produce Safety Rule addresses the mitigation of risks of contamination from domesticated animals and wildlife during the growing, harvesting, packing, and handling of fresh produce covered by the regulation (6, 7).

Wild bird populations are widely recognized as potential causes of significant economic losses due to crop damage (8), and they pose a potential public health concern due to foodborne pathogen carriage. Whereas large mammals can be kept out of produce fields by adequate fencing, growers have expressed concerns about the challenges of minimizing wild bird intrusions and the subsequent potential for pathogen contamination of produce, soil, and water. Growers and food safety specialists alike have been unable to define a risk-based action threshold for bird intrusion related to numbers of individuals. In the FDA’s recently released draft guidance (6), Canada geese (Branta canadensis) and other bird species intrusions are used as examples to illustrate science-based minimum standards related to reducing potential food safety risks from domesticated or wild animals during fresh produce production; however, these standards are not prescriptive and require careful monitoring and decision-making by the farmers and food safety professionals. The intrusion into produce fields by bird species that form large flocks, e.g., the European starling (Sturnus vulgaris), the American crow (Corvus brachyrhynchos), or some blackbird species (Icteridae), likely results in a higher probability and amount of fecal contamination of produce (6). Many birds perch on sprinklers to drink water, and small birds like sparrows also hop and perch on leafy green plants. It is known that birds can shed foodborne pathogens, such as Shiga toxin-producing Escherichia coli (STEC) (E. coli O157:H7 included) and *Salmonella* spp. (9, 10) in their feces. Fecal contamination is considered the primary risk of introduction of enteric zoonotic pathogens from animals into the produce environment, but there is a need to assess other routes of transmission. For example, Campylobacter jejuni was cultured from oral swabs from feral pigs, suggesting a potential risk for foodborne pathogen transmission during foraging (11). It has also been speculated that carriage of foodborne pathogens in the oral cavity and the exterior (feet and feathers) of birds may pose a risk.

The proximity to a cattle operation is considered an additional risk factor for produce contamination, as cattle are a common host of foodborne pathogens (12, 13). On cattle operations, birds intermingle with cattle and can acquire foodborne pathogens from the animals and the contaminated environment or feed, subsequently acting as pathogen vectors. For example, it has been found that European starlings can transmit E. coli O157:H7 between dairy farms (14), and a European starling sampled on a farm carried E. coli O2:H29 indistinguishable from E. coli O2:H29 found in cattle on the same site (15).

Despite the large diversity of avian taxa, a few bird taxonomic groups and species have received most attention for foodborne pathogen research. Recent reviews highlight that published research on foodborne pathogens in birds in agricultural land is scarce and focused on a few species (8, 16), which is not representative of the diverse avian community that can be found in agricultural landscapes. Geese and swans (17) with particular emphasis on Canada geese (18), European starlings, house sparrows (Passer domesticus), and feral pigeons (Columba livia) are often the main targets of foodborne pathogen studies and reviews, especially urban populations of those species (19). Thus, there are gaps in knowledge regarding the carriage of foodborne pathogens by many avian species present and abundant in agricultural lands and particularly by those species associated with riparian habitats. For years, there has been a controversy that farmers may remove noncrop vegetation for food safety reasons, a practice that threatens riparian habitats (5, 20). Thus, studies are necessary to inform policy and conservation strategies within agricultural lands that are compatible with the microbiological safety of produce. Relatedly, recent efforts on many U.S. produce farms strived to balance food safety and habitat conservation.

This repeated cross-sectional study aimed to actively survey the year-round shedding of *Salmonella* spp., E. coli O157:H7, and non-O157 STEC by avifauna associated with riparian habitats located within agricultural fields in California. The specific study sites were an operating produce commercial ranch (CR) and a national wildlife refuge (NWR), both selected because of their location in a major agricultural area of California, the presence of surface water, and an associated riparian habitat, bird abundance, and proximity to free-range cattle. Further objectives were to elucidate whether birds carry these pathogens in their oral cavity and/or external parts (feet and feathers) and to determine by subtyping methods whether wild birds and sympatric free-range cattle share the same pathogen strains.

The originality of this study resides in its experimental design, involving constant, yearlong sampling effort at two specific sites, the study location being on a commercial farm, and the screening of a diversity of avian species.

## RESULTS

### Bird capture success and total sample size.

At an approximate rate of two sampling days per month, per the two study sites, a total of 60 avian species were sampled during the study period. With mist nets and ground traps, 583 birds of 57 species were successfully captured, individually sampled, and tested for the targeted foodborne pathogens. From most birds, multiple samples were taken and processed separately (feces and cloacal swabs, foot/feather swabs, and oral swabs). Briefly, the final set of individual bird samples comprised the following taxonomic groups: sparrows (*n* = 273), icterids (*n* = 64), woodwarblers (*n* = 58), tyrant flycatchers (*n* = 42), kinglets (*n* = 24), thrushes (*n* = 21), mimids (*n* = 14), chickadees (*n* = 14), game birds (*n* = 11), grosbeaks (*n* = 10), finches (*n* = 7), swallows (*n* = 6), woodpeckers (*n* = 6), wrentits (*n* = 6), wrens (*n* = 6), corvids (*n* = 4), doves (*n* = 3), vireos (*n* = 2), buntings (*n* = 1), wading birds (*n* = 1), hawks (*n* = 1), and kingfishers (*n* = 1). Table S1 in the supplemental material shows in detail the species and number of individuals per species captured and sampled from each study site, whereas the scientific names of the bird species can be found in Table S2. Opportunistically collected bird samples (pooled feces) belonged to three additional species, Canada geese (*n* = 16), greater white-fronted geese (Anser albifrons, *n* = 10), and sandhill cranes (Antigone canadensis, *n* = 6).

A total of 1,369 bird samples were collected and processed for pathogen detection. Specifically, 615 fecal samples were collected, with 234 individual fecal samples from the CR, 349 individual fecal samples from the NWR, and 32 pooled fecal samples from the NWR (which corresponded to bird convenience samples). Of the 401 foot/feather swabs processed, 188 swabs were collected from the CR and 213 swabs from the NWR. With regard to the 353 individual oral swabs processed, 156 swabs were gathered on the CR and 197 swabs in the NWR.

### Overall prevalence of foodborne pathogens in birds.

Low rates of *Salmonella* spp., E. coli O157:H7, and non-O157 STEC were detected in birds, mostly from fecal samples (Table 1). Interestingly, two birds were positive for non-O157 STEC cultured from their feet/feathers. Eight of the 60 bird species screened were positive for foodborne pathogens (13.3%; Table 2). The red-winged blackbird (Agelaius phoeniceus) was the only bird species found to be positive for more than one pathogen (STEC O26 and *Salmonella* spp.), but none of the individuals sampled in this study carried more than one pathogen.

**TABLE 1:** Number of samples tested, positive samples, and pathogen prevalence

Sample group	No. of samples	No. (%) of positive samples of:		
		*Salmonella*	E. coli O157:H7	Non-O157 STEC
Individual fecal samples^*a*^	583	3 (0.5)	2 (0.34)	3 (0.5)
Pooled fecal samples	32	0	0	4 (12.5)
Individual oral swabs	353	0	0	0
Individual feet/feathers swabs	401	0	0	2 (0.5)

a For 501 birds, the fecal sample was combined with an individual cloacal swab.

**TABLE 2:** Pathogen-positive bird species and frequency of pathogen carriage in any sample type by location

Bird species	No. of samples from CR/NWR^*a*^	Pathogen(s) found	Frequency (%) of carriage in CR/NWR (no. of positive samples)^*b*^
Song sparrow	25/16	*Salmonella* Newport	4 (1)/0
Golden-crowned sparrow	10/47	STEC O26	10 (1)/0
Savannah sparrow	14/1	*Salmonella* III 17:g,z51:-	7.1 (1)/0
Red-winged blackbird	26/4	STEC O26	3.9 (1)/0
		*Salmonella* Saugus	3.9 (1)/0
Bewick’s wren	0/4	STEC O103	NC/25 (1)
Ruby-crowned kinglet	1/23	E. coli O157:H7	0/8.70 (2)
Canada geese^*c*^	0/16	STEC O163	NC/12.5 (2)
Greater white-fronted geese^*c*^	0/10	STEC O26, O84	NC/20 (2)

a CR, commercial ranch; NWR, national wildlife refuge.

b NC, no pathogen carriage because this species was not captured/sampled at this study site.

c Pooled samples. The number of positives does not correspond to individuals.

### E. coli O157 and non-O157 STEC in birds.

**FIG 1:**
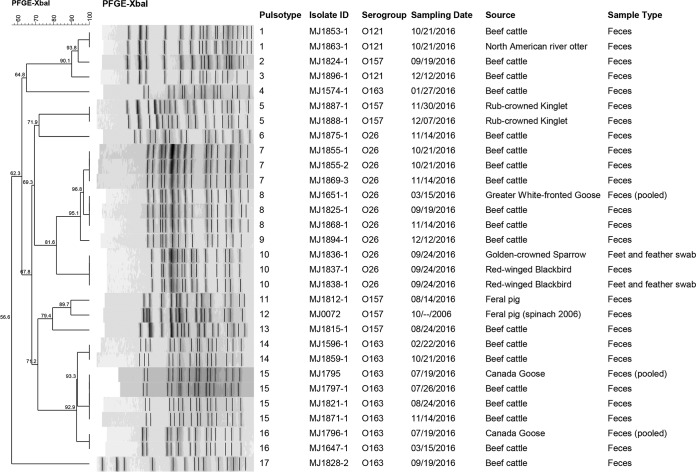
Dendrogram of STEC isolates from cattle, birds, and other wildlife in northern California, 2015 to 2016.

At the CR, two of 234 (0.85%) birds were positive for non-O157 STEC, including one male red-winged blackbird (1/26 [3.85%]) and one golden-crowned sparrow (1/10 [10%]) (Zonotrichia atricapilla). The former was positive in both fecal and foot/feather samples, whereas the latter was positive in feet/feathers only. Both birds were sampled on the same day at the end of September. All of these isolates belonged to the STEC serogroup O26 and had the same virulence factors (Table 3). By pulsed-field gel electrophoresis (PFGE), these three isolates showed 100% similarity (pulsotype 10, Fig. 1). STEC O26 isolates obtained from cattle or birds from the NWR study site were not clustered in this pulsotype.

**TABLE 3:** Serogroup and virulence factors of E. coli O157 and non-O157 STEC bird isolates

Mo	Location	Bird species	Sample type	Gene presence/absence^*a*^					Serogroup or serotype
				*hlyA*	*eaeA*	*stx*_2_	*stx*_1_	*ehxA*	
March	NWR	Greater white-fronted goose	Pooled feces	0	0	0	1	1	O26
				1	1	0	1	1	O84
July	NWR	Canada goose	Pooled feces	1	0	1	1	1	O163
				1	0	1	1	1	O163
September	CR	Red-winged blackbird (same individual)	Feces	1	1	0	1	1	O26
			Feet and feathers	1	1	0	1	1	O26
		Golden-crowned sparrow	Feet and feathers	1	1	0	1	1	O26
November	NWR	Bewick’s wren	Feces	1	1	0	1	1	O103
		Ruby-crowned kinglet	Feces	1	1	1	1	1	O157:H7
		Ruby-crowned kinglet	Feces	1	1	1	1	1	O157:H7

a 0, absent; 1, present.

At the NWR, 12.5% (4/32) of the pooled bird fecal samples were positive for non-O157 STEC. These were collected from greater white-fronted geese sampled in March (serogroups O26 and O84) and Canada geese sampled in July (serogroup O163). There was no other positive bird sample found until the end of November. The feces of a Bewick’s wren (Thryomanes bewickii) was positive (1/4 [25%]) for O103 STEC. On the same day, the fecal sample of a ruby-crowned kinglet (Regulus calendula) was positive for E. coli O157:H7. One week later, another ruby-crowned kinglet fecal sample was positive for E. coli O157:H7 (2/23 [8.70%]). These two O157:H7 strains showed 100% similarity by PFGE (Fig. 1).

### *Salmonella* spp. in birds.

Three birds were positive for *Salmonella* spp. over the entire study period (Table 2), and they were positive in fecal samples only. All three *Salmonella* carriers were mist netted and sampled on the CR. With regard to their age, two of them were in their hatch year (the song sparrow [Melospiza melodia] and savannah sparrow [Passerculus sandwichensis]), and one was a male adult (red-winged blackbird).

### Factors explaining pathogen carriage in birds.

Because of the low overall prevalence of pathogens, we had to consider the carriage of all three pathogens combined as a binary response variable (positive to at least one pathogen versus negative to all three pathogens). As explained in Materials and Methods, we tested the associations between pathogen carriage and each of the explanatory variables for those bird taxonomic groups that were sufficiently abundant on both sites (sparrows, 273; icterids, 64). In particular, in sparrows, we tested whether site, season, or age had an effect on pathogen carriage. In icterids, we tested the effects of site, season, or sex.

Using Fisher’s exact test, for sparrows, we found no effect of site (*P* = 0.15) or age (hatch year versus older, *P* = 0.23) on the carriage of pathogens. Season had a significant effect (*P* = 0.04), with positive sparrows being detected only in spring and summer. In icterids, we found no effect of site (*P* = 0.2), season (*P* = 0.06), or sex (*P* = 0.45).

### Pathogens in the NWR cattle and subtypes shared with birds.

Table 4 shows the monthly prevalences of pathogens in the cattle herd tested from the NWR and concurrent findings in birds at the two sampling locations. No cattle sample was positive for *Salmonella* species. E. coli O157:H7 was absent most of the year, with an overall prevalence of 5% and a monthly prevalence ranging from 0% to 5% (mostly detected in late summer and fall). The only time where E. coli O157:H7 was found in birds was the end of November, but cattle were negative for this pathogen at that time and at the following sampling event. Furthermore, the PFGE showed that E. coli O157:H7 was not shared between cattle and sympatric birds, and cattle and bird isolates belonged to different and distant pulsotypes (pulsotype 5 in birds but pulsotypes 2 and 13 in cattle, with similarities ranging from approximately 60% to 70%; Fig. 1).

**TABLE 4:** Monthly prevalences of pathogens in cattle feces (NWR herd) and concurrent pathogen findings in birds^*a*^

Mo	No. of cattle samples	No. (%) of:			Non-O157 serogroup(s)	Bird findings
		*Salmonella* spp.	E. coli O157:H7	Non-O157 STEC		
January	15	0	0	3 (20)	O163	Negative
February	22	0	1 (4.5)	9 (40.9)	O163, O26	Negative
March	30	0	0	8 (26.7)	O163, O26, O136	O26 and O84 in the NWR
April	36	0	0	15 (41.7)	O136	*Salmonella* sp. in CR
May	18	0	0	8 (44.4)	O121, O145	Negative
June	22	0	0	1 (4.5)	O136	Negative
July	20	0	0	1 (5)	O163	O163 in the NWR
August	20	0	1 (5)	6 (30)	O91, O163	*Salmonella* sp. in CR
September	20	0	1 (5)	11 (55)	O26, O121, O163	*Salmonella* sp. and O26 in CR
October	22	0	1 (4.5)	11 (50)	O26, O121, O163, O178	Negative
November	20	0	0	11 (55)	O26, O163	O103 and O157:H7 in the NWR
December	20	0	0	13 (65)	O26, O121	Negative

a CR, commercial ranch; NWR, national wildlife refuge.

In cattle, non-O157 STEC was detected each month, with large fluctuations (from 4.5% to 65%). In birds, this pathogen was detected much less frequently (March, July, September, and November). No clear pattern of seasonality nor a link to STEC prevalence in cattle could be observed. PFGE grouped the O26 isolates from the NWR site in four clusters, pulsotypes 6 to 9. From these pulsotypes, only pulsotype 8 was common to cattle and birds (greater white-fronted geese). This pulsotype was first isolated from geese and then on two later sampling events in the fall from cattle.

Serogroup O163 was also found in cattle as well as in birds in the NWR, in resident Canada geese in particular. PFGE grouped the O163 isolates from this site in five clusters, pulsotypes 4 and 14 to 17. From these, only pulsotypes 15 and 16 were common to cattle and resident Canada geese. Pulsotype 15 was concurrently isolated in July from the two host species. On two later dates during the summer and fall, cattle were again positive for this pulsotype. In contrast, pulsotype 16 was first isolated from cattle in March and from resident Canada geese in July but was no longer detected in any species of the NWR.

### Pathogens in nonbird scat samples.

Table 5 shows the results of pathogen detection in nonbird scats opportunistically collected at the sampling sites. In spring, a lizard and a rabbit from the CR carried Salmonella enterica serotype Muenchen. On the CR, Salmonella enterica serotype Newport was isolated from a feral pig sample, and another feral pig sample collected on the same day was positive for E. coli O157:H7. This isolate was compared using PFGE to the feral pig strain that was associated with the 2006 spinach-related outbreak traced to the same region (3); they belonged to different pulsotypes (11 and 12), displaying 89.7% similarity. On the NWR, a North American river otter was positive for STEC O121, the same pulsotype that was isolated on the same sampling day from sympatric cattle (pulsotype 1, Fig. 1).

**TABLE 5:** Nonbird convenience samples and pathogens found

Season	Location^*a*^	Common name (*n*)	Scientific name	Pathogen(s) detected (no. of positive samples)
Spring	CR	Western fence lizard (2)	*Sceloporus occidentalis* (suspected)	*Salmonella* Muenchen (1)
		Rabbit (1)	*Sylvilagus* sp.	*Salmonella* Muenchen (1)
		Black-tailed deer (3)^*b*^	*Odocoileus hemionus*	Negative
	NWR	Coyote (1)	*Canis latrans*	Negative
Summer	NWR	American river otter (1)	*Lontra canadensis*	Negative
	CR	Feral pig (6)^*b*^	Sus scrofa	E. coli O157:H7 (1), *Salmonella* Newport (1)
Fall	NWR	American river otter (1)	*Lontra canadensis*	STEC O121 (1)

a CR, commercial ranch; NWR, national wildlife refuge.

b Pooled samples. The number of positives does not correspond to individuals.

## DISCUSSION

### Prevalence and dynamics of foodborne pathogens in wild birds.

For research studies conducted on agricultural lands, the engagement of the industry is essential. However, it can be difficult to locate an industry partner as highly committed as needed for such studies. In general, research of foodborne pathogens in birds is scarce compared to that in rodents and other wildlife, probably because of the additional difficulty in capturing and sampling birds and because of the permits and expertise required.

The non-O157 STEC prevalence in bird feces identified in this study (0.5%) is less than that found by Rivadeneira et al. (21) in birds sampled from leafy greens fields in proximity to a concentrated animal feeding operation (CAFO) in southern Arizona (4.9%). However, other studies that examined STEC fecal shedding in wild bird populations have found low prevalences, similar to our results, despite different isolation methods and therefore not being directly comparable. For example, Nielsen et al. (15) reported 4 out of 244 (1.64%) STEC-positive birds mist netted at Danish farms, but their sampling occurred during only 1 week in summer. In the Czech Republic and Austria, Konicek et al. (22) sampled the cloacae of 1,191 birds from numerous locations, but only 2 (0.17%) carried STEC (unspecified songbird and waterfowl species).

The fact that STEC was isolated from the exterior (feet/feathers) of wild birds raises further questions regarding STEC transmission and contamination. The feathers and feet of wild birds can harbor a variety of bacteria, as they frequently contact the environment. In fact, feather swabs from chickens and ducks at a Canadian petting zoo were positive for STEC O103 and O26 (23). However, STEC and other enteric pathogens are most often isolated from bird feces. Out of 223 birds sampled, Borges et al. (24) found three birds carrying enteropathogenic Escherichia coli in their oral cavity in Brazil (two urban pigeons and one white-eyed parakeet [Psittacara leucophthalmus]). Unlike these authors, we did not find any wild bird oral swab positive for pathogens. It is unknown whether the differences in isolation methods may explain this outcome. In our sample set, the overall prevalence of birds carrying STEC in feces was low and was very rare in feet/feathers. Nevertheless, we found some STEC serogroups that are highly important for public health: O26 and O103 are the second and third most frequently cultured serogroups from human cases in the United States (25). In California in particular, O26 is by far the most frequently reported serogroup, with O103 being the third most common (25).

At NWR, we found two ruby-crowned kinglets positive for E. coli O157:H7 within a week on the same mist netting spot. In contrast, E. coli O157:H7 was not detected in birds at CR. It is noteworthy that in spite of E. coli O157:H7 being carried by feral pigs near CR, and probably being present in the environment and in other wildlife, no bird in this study site was positive for this pathogen. In a previous survey conducted within the California central coast near CR, Gordus et al. (26) tested 871 small birds for E. coli O157:H7 and found only one positive bird, a dark-eyed junco (Junco hyemalis). Concurrent testing of beef cattle adjacent to CR would have been interesting for comparison, but we did not have landowner permission.

In light of our results, some bird species may be more prone than are others to carry STEC, probably due to their inherent behaviors. The golden-crowned sparrows forage on the ground, where they spend most of their time. Red-winged blackbirds are also ground foragers; they breed within wet places, like marshes, and are known for their association to cattle and other livestock. Rivadeneira et al. (21) also found a *Salmonella* sp. and STEC in red-winged blackbirds (1/66 and 2/66, respectively) in a leafy green production area in southern Arizona. However, our finding of STEC in Bewick’s wren and ruby-crowned kinglets is surprising, as they feed on insects located on surface of leaves and branches. Whereas we could suspect that their insectivorous diet is related to this pathogen finding, many other birds in our sample are mainly insectivorous (flycatchers, woodpeckers, thrushes, etc.), and they were negative for STEC. The Bewick’s wren and the ruby-crowned kinglet inhabit bushes and rarely venture into open areas, like produce fields; thus, in our opinion, they do not constitute a high risk for produce contamination. Given that few bird species and individuals appear to be STEC carriers, the potential contamination of soil and water in agricultural lands by birds may be seasonal and dependent on the bird species present, their abundance, and their migration patterns. For example, we found the greater white-fronted geese to be positive for STEC in March, right before this species left the NWR to migrate to their breeding grounds in the Arctic. Golden-crowned sparrows and ruby-crowned kinglets are also migrants that spend winter months in our study sites, whereas the rest of bird species found positive for pathogens are year-round residents in California (with the exception of some subspecies of song sparrow and savannah sparrow). Canada geese in particular are both, as a small flock is resident in the NWR, but large numbers gather in this site in winter.

Despite the low prevalence in birds found by this study, our repeated cross-sectional sampling scheme enabled the detection of pathogen shedding that resembles clusters of infection, or in other words, an “outbreak-like” pattern. In the case of STEC O26, the fact that two different bird species were carrying the same pulsotype suggests a common source of contamination on or near the produce ranch (CR) by the end of summer (September). The carriage of this pulsotype on bird feet/feathers in addition to being shed in feces suggests that the environment, for example, an on-farm sedimentation pond, may be contaminated with this pathogen. Similarly, the finding of the same E. coli O157:H7 pulsotype in two birds of the same species sampled on the same site 1 week apart suggests a common source of contamination in the environment. Some of the reasons explaining these cluster-like events could be intermittent and/or seasonal shedding by birds in their feces, episodes of higher contamination of the environment, or a higher exposure of birds to pathogens due to a change in behavior.

Overall, bird samples had a low prevalence of *Salmonella* spp. (0.5%), and *Salmonella* spp. were not detected in any cattle fecal sample. Likewise, Gorski et al. (27) found a *Salmonella* sp. in only 1 of 795 (0.13%) cattle samples in a longitudinal survey conducted in Monterey County, a major produce region of California that neighbors one of our study sites (CR).

*Salmonella* spp. were only isolated from the CR, both from birds and from other wildlife. This suggests that the CR environment may be contaminated with this pathogen to a wider extent than the NWR area. Despite the low prevalence, our finding of *Salmonella* serotypes of public health concern is important. One bird trapped on the CR carried *Salmonella* Newport, which is among the top three serotypes causing outbreaks in the United States, with an increased number of cases in 2015 in comparison to 2005 (28, 29). Salmonella enterica serotype Saugus has been previously isolated from deer scat in the neighboring Monterey County (27) and is also responsible for human cases of salmonellosis (28, 29). Despite Hughes et al. (30) finding that the fecal carriage of *Salmonella* bacteria is a risk factor for isolating E. coli with the *stx*_2_ gene in wild birds, none of the individuals tested by us were concurrently carrying more than one pathogen.

### Avian diversity near produce fields.

The results of the present study show the diversity of bird species that can be found in a riparian habitat within agricultural lands (Table S1). On a produce ranch in neighboring Monterey County, Navarro-Gonzalez and Jay-Russell (31) performed count transects to describe the diversity of bird species that use and feed in produce fields. Some of these species coincide with the species captured during the present study (Table S1). During the study period, we have observed the presence of savannah sparrows, house finches, red-winged blackbirds, and great-tailed grackles within lettuce and broccolini fields at the CR. In fact, house finches, white-crowned sparrows, and golden-crowned sparrows are known lettuce pests that cause crop damage (32).

Nevertheless, the impact of abundance and diversity of birds in agricultural land needs to be considered holistically by weighing the risks and benefits of avifauna. Many birds are insectivorous, and their feeding activity can provide pest control services for the grower (33). Likewise, rodent control provided by avian predators, such has barn owls (Tyto alba), can be particularly beneficial for agriculture (34, 35). Other known services contributed by birds are soil fertilization and pollination. In fact, Karp et al. (36) found that produce from areas surrounded by natural vegetation (including riparian habitats) were not more contaminated with enterohemorrhagic E. coli (EHEC) than were fields surrounded by cropland or grazeable land. The results of our study also support the compatibility of comanaging conservation and food safety practices for fresh produce production.

### Pathogen subtypes shared by cattle, birds, and other wildlife.

This study focused on wild birds primarily captured in mist nets. Overall, a limited sharing of foodborne pathogens between cattle and wildlife was found, especially avian species. Concurrent prevalence of foodborne pathogens in cattle did not explain the pathogen findings in birds (Table 4). The detection of E. coli O157:H7 in cattle (February and August through October) was not associated with the detection of this pathogen in sympatric birds. Similarly, findings of non-O157 STEC in birds did not consistently coincide with peaks of non-O157 STEC prevalence in the NWR cattle.

Specifically, E. coli O157:H7 strains were not shared between cattle and wild birds at the NWR, suggesting that birds examined at this location and time did not represent a reservoir of STEC subtypes associated with cattle. Interestingly, the only STEC matches by PFGE (O26 and O163) between birds and cattle were isolated from convenience samples from wild geese. Wild geese are known to be carriers of pathogenic E. coli, including STEC (37, 38), and a risk factor for O157 exposure in cows (39). Additionally, carriage of the same STEC strain occurred in cattle, as well as in a North American river otter (Fig. 1) for which water was possibly involved as a transmission vehicle.

The fact that *Salmonella* Muenchen was found in a lizard sample and a rabbit sample on the same site and during the same season suggests that this serotype was widespread in the environment. However, no bird was found carrying this serotype at any point during this study.

### Limitations of this study.

This study aimed to monitor the dynamics of foodborne pathogens in wild birds residing near agricultural lands during 1 year, but there may be a year-to-year variability. A multiyear study would be needed to observe temporal patterns, both in bird diversity and abundance as well as in the ecology of foodborne pathogens. In fact, with a 3-year study on wild bird feces, Hughes et al. (30) found that the proportion of E. coli strains carrying virulence genes varied significantly from year to year. To better understand wildlife-livestock interactions and risks of pathogen dissemination, more cattle operations should be studied. Other limitations encountered were meteorological factors such as wind and rain, which prevented us from mist netting or even from accessing a study site. In 2016, winter months were considerably rainy, and thus, our winter sample size is smaller than that in other seasons. However, it is a substantial sample size considering that most bird banding stations do not operate in winter due to the low capture success and the difficulties posed by weather.

### Conclusions.

This study provides evidence that most bird species, and those species associated with riparian habitats in particular, do not often carry foodborne pathogens. However, even low prevalence can represent risk for contamination if the bird populations aggregate in large groups or produce copious amounts of fecal material. Seasonal and shedding factors as well as migration patterns may also promote a higher risk of contamination than that with an overall low average prevalence observed in this and other studies. We consider that the best measure for producers to minimize the risk of microbial contamination from bird intrusions into raw crops such as leafy greens is to not harvest heavily contaminated parts of fields and provide training that instructs harvesters not to harvest leafy greens visibly contaminated by feces, including bird droppings and crop damage. This study supports the existing agricultural industry practice of having trained harvest operation scouts walking ahead of mechanized harvest machines for tender greens crops, such as baby spinach. As described in the FDA’s draft guidance (6), monitoring for potential microbial contamination risks from domesticated animals and wildlife is crucial to protect the consumers. For example, special care should be taken in produce fields where large groups of geese and other flocking birds aggregate and forage. The fact that birds can carry STEC on their feet or feathers deserves further attention, and more research is needed to elucidate if they can contaminate irrigation water systems and other crop inputs and equipment.

To advance our knowledge regarding the carriage of foodborne pathogens by birds on agricultural land, future research should target those species that specifically forage on produce fields in large flocks and that have been found to be positive for pathogens by this and other studies.

## MATERIALS AND METHODS

### Study sites.

Two sites in northern California were selected based on the following criteria: (i) geographic location within predominant agricultural production areas of California, (ii) presence of surface water and riparian habitats, and (iii) immediate adjacency to rangeland raising beef cattle.

One of the study sites was a produce commercial ranch (CR) in San Benito County, on the central coast foothills of California. The CR was located by the San Benito River in a valley immediately adjacent to the Salinas Valley. San Benito County has a history of produce contamination and high risk factors for contamination. In fact, the above-mentioned 2006 spinach E. coli O157:H7 outbreak was traced to this county. The pathogen is thought to be widespread in the environment due to fecal contamination of the soil and water by cattle. The CR where bird sampling took place is a nearly 500-acre irrigated cropland for organic raw crop production (mainly lettuce and broccolini). Immediately adjacent to the produce fields, a ranch operates a beef cattle herd on 7,000 acres following rotational grazing principles.

The other study site was the Stone Lakes National Wildlife Refuge (NWR) in Sacramento County, on the delta of the California’s Central Valley. This national wildlife refuge (6,550 acres) is located on the Stone Lakes Basin floodplain and also serves as a buffer for urban encroachment into the delta, where land use is predominantly agricultural. In the NWR, a herd of approximately 100 cows is kept outdoors year round and managed according to rotational grazing principles. In summer, the cattle graze on irrigated pasture. We were permitted to collect fecal samples from the cattle herd present on the NWR, but we did not have access to the land adjacent to the CR (private property).

### Bird mist netting and trapping.

Forty-four bird sampling events took place over a year, from November 2015 to December 2016. Either 9 or 10 nets were used during each sampling event. Mist netting was supplemented by ground traps at each site. Mist nets and ground traps were placed in proximity to a water body (reservoir or lake). In total, the sampling effort was 1,285 “net hours” and 177 “trap hours.” Birds were individually identified with an aluminum band provided by the USGS Bird Banding Laboratory. All mist netting, trapping, and banding were performed by author S.W. (federal bird banding permit number 22853 and California scientific collection permit number 2994). More details on the mist netting and trapping procedures and their capture success rates can be found in the supplemental material.

### Collection of bird samples.

All sampling procedures were approved by the UC Davis IACUC (protocol number 18904) and the permits mentioned above held by the author S.W. Birds caught in the nets or traps were carefully removed and placed in previously autoclaved paper bags for transport to a nearby area with appropriate protection from the weather to process them. Birds were kept inside the paper bags while waiting to be banded and sampled, often defecating inside. Each paper bag containing individual feces was placed in a sterile Whirl-Pak bag and moisturized with 9 ml of phosphate-buffered saline (PBS) in the field. In addition, a cloacal swab (CultureSwab with Amies gel; Fisher BD, Sparks, MD) was used for each bird, with the exception of very small birds (such as kinglets) and birds in obvious distress. The oral cavity of birds was sampled with a Fisher BD CultureSwab with Amies gel. Feet and feathers from the ventral area were swabbed with a sterile Fisher cotton ball applicator. After collection of the foot/feather swab, the applicator was placed in a 10-ml tube with 3 ml of PBS, the wood handle was broken off to avoid contamination, and the tube was capped. During handling and sampling, bird handlers followed good hygiene practices to avoid contamination of the samples and contamination between birds. Hand sanitizer and a 70% ethanol spray to disinfect surfaces and tools (e.g., scales) were used after a bird defecated or left feathers behind.

All samples were stored on ice and taken directly to the laboratory for processing within 24 h of sample collection. On the NWR, flocks of birds that defecated on the cattle pasture were opportunistically sampled. Three to five fresh feces samples were gathered from the ground and pooled into a sterile Whirl-Pak bag.

### Collection of cattle feces.

Access to the NWR to collect cattle feces was granted by the National Wildlife Refuge System special use permit number 1603. Cattle were sampled monthly from January to December 2016. In total, 260 fresh fecal samples from cattle were collected from the ground and placed into sterilized cups (National Scientific, Claremont, CA) using sterile scoops (Bel-Art, Wayne, NJ). On average, 22 samples were collected at each event. As cattle rotated into different pastures, the location of each sampling event was recorded. Samples were stored on ice and taken to the laboratory for processing within 3 h of sample collection.

### Convenience sampling of nonbird scat.

Although not specifically targeted in this study, scats from nonbird wildlife were opportunistically collected when found at the mist netting sites or on the cattle pasture in the NWR. Fecal samples were identified with the use of a field guide for tracks and scat identification specific to California wildlife (40). The species of origin was preliminary identified based on the scat morphometrics and other signs, such as tracks; to increase our certainty, we cross-validated our observations with local knowledge and daily observations from the CR and NWR staff. These samples provided additional information about the pathogens present in the environment and other animal hosts at our study sites, and they aid in the interpretation of the role of wild birds as reservoirs of foodborne pathogens. Table 5 shows the number of samples, species of origin, and the site and season where nonbird convenience samples were collected.

### Detection of STEC in birds, cattle, and others.

Bird individual fecal samples were processed in combination with their corresponding cloacal swab. Bird oral swabs and foot/feather swabs were processed individually. All bird individual samples were preenriched by placing the swab or cloacal swab plus feces into 9 ml tryptic soy broth (TSB; BD Diagnostic Systems, Sparks, MD). Samples were then incubated for 2 h at 25°C with agitation at 100 rpm, followed by 8 h at 42°C with agitation, and held overnight at 6°C, using a Multitron programmable shaking incubator (Eppendorf, Hauppauge, NY). The same procedure was followed with 10 g of fecal material to detect EHEC/STEC in cattle, pooled bird samples (geese and cranes), and nonbird wildlife.

For the detection of E. coli O157, immunomagnetic separation (IMS) using Dynal anti-E. coli O157 beads (Invitrogen/Dynal, Carlsbad, CA) was performed on 1 ml TSB enrichment broth with the automated Dynal bead retriever (Invitrogen), per the manufacturer’s instructions (41). After incubation and washing, 50 μl of the resuspended beads was plated onto Rainbow agar (Biolog, Hayward, CA) with novobiocin (20 mg/liter) and tellurite (0.8 mg/liter) (MP Biomedicals, Solon, OH). Fifty microliters of the resuspended beads was also plated onto MacConkey II agar with sorbitol supplemented with potassium tellurite (2.5 mg/liter) and cefixime (0.05 mg/liter) (CT-SMAC); plates were streaked for isolation and incubated for 24 h at 37°C. Suspect E. coli O157 isolates were confirmed using traditional PCR for the *rfbE* gene (42).

To detect non-O157 STEC, 1 ml of enriched TSB was transferred into 9 ml modified EHEC (mEHEC) broth (Biocontrol, Bellevue, WA) and then incubated for 12 h at 42°C, followed by plating and incubating on CHROMagar STEC (DRG International, Inc., Springfield, NJ). The mEHEC broth enrichment was adapted in our laboratory for fecal and swab samples using a protocol developed for EHEC recovery from spinach leaves under natural conditions (43). Up to 6 presumptive STEC positive colonies were confirmed for the presence of *stx*_1_ and/or *stx*_2_ genes by real-time PCR (Eppendorf, Hauppauge, NY), as in reference 41. Confirmed STEC isolates were assigned an isolate identification (ID) comprising the sample identification and a dash representing the individual colony pick (e.g., MJ1853-1). STEC isolates were then characterized for virulence genes (*stx*_1_, *stx*_2_, *eaeA*, *hlyA*, and *fliC*) using conventional PCR (42). All isolates from bird origin were submitted to the Penn State University E. coli Reference Center for O serogrouping (University Park, PA). Cattle isolates were selected for O serogrouping based on the sampling date and the pattern of virulence factors to represent the monthly diversity of serogroups in the cattle herd.

### STEC investigation by pulsed-field gel electrophoresis.

Strains of the same serogroup with the same virulence factors from different animal species were further investigated by PFGE to elucidate if they were attributable to a common source. For comparison, the archived E. coli O157:H7 strain isolated from feral pigs during the 2006 outbreak investigation was included. In total, 30 STEC isolates were analyzed using the Centers for Disease Control and Prevention protocol for PFGE (44). PFGE followed XbaI digestion of selective STEC isolates in the presence of bovine serum albumin (BSA). Gels were run for 17 h and 30 min with E. coli settings (Bio-Rad Chef Mapper XA). A dendrogram was constructed using the unweighted pair group method using average linkages (UPGMA) algorithm and Dice coefficiency with 1.5% tolerance (BioNumerics v 7.1).

### Detection of *Salmonella* spp. in birds, cattle, and others.

Individual bird fecal samples were processed in combination with their corresponding cloacal swab. Bird oral swabs and foot/feather swabs were processed individually. After preenrichment, *Salmonella* spp. were recovered by adding 100 μl of enriched buffered peptone water to 10 ml Rappaport-Vassiliadis (RVS) broth (BD Becton, Sparks, MD) and incubating for 48 h at 42°C, as described previously (45). A loopful of RVS bacterial suspension was then be streaked onto xylose-lysine-Tergitol 4 (XLT4) agar plates and incubated for 24 to 48 h at 37°C for isolation. Up to six suspect colonies per positive plate were confirmed by PCR (46) and stored on a cryogenic medium (TSB with 15% glycerol; Fisher Scientific, Pittsburgh, PA) at −80°C. The same procedure was followed with 10 g of fecal material to detect *Salmonella* spp. in cattle and nonbird convenience samples.

### Statistical analyses.

Potential explanatory variables considered for pathogen presence outcome included bird taxonomic group, individual sex and age (for those bird species where it was possible to determine), site, and season. In light of the capture success by bird taxonomic groups and study site, we built contingency tables to test specific hypotheses. In particular, the group “sparrows” and the group “icterids” were present and sampled in both study sites and yielded positive pathogen results. Sex was not determinable in the species that mainly represented the group sparrows; thus, we tested whether site, age, or season had an effect on the carriage of pathogens. However, sex could be determined in icterids and was used as an explanatory variable, as well as site and season. In the icterid group, the effect of age could not be tested, because all individuals except for one were in the category “after hatch-year.” By bird group, we tested the association of pathogen carriage versus each of the applicable explanatory variables with multiple Fisher’s exact test implemented with the R software 3.4.0 (47).

## Supplementary Material

Supplemental file 1
