# Supplementary material for: Carriage and Subtypes of Foodborne Pathogens Identified in Wild Birds Residing near Agricultural Lands in California: a Repeated Cross-Sectional Study
Source: Appl Environ Microbiol. 2020 Jan 21;86(3):e01678-19. doi: 10.1128/AEM.01678-19 (PMC6974635; doi:10.1128/AEM.01678-19)
Supplement: Supplemental file 1 [file zam003209576s1.pdf]

Supplemental Information Table 1. Group and species of birds individually captured and sampled by location (CR= Commercial Ranch, NWR= National Wildlife Refuge)

Group and species	Nº of birds sampled in CR/NWR
Sparrows and their allies:	
White-crowned sparrow*	75/ 15
Song sparrow*	25/ 16
Lincoln's sparrow	13/ 1
California towhee*	5/ 1
Golden-crowned sparrow	10/ 47
Lark sparrow	1/ 0
Fox sparrow	1/ 11
Spotted towhee	1/ 16
Savannah sparrow*	14/1
Dark-eyed junco (Oregon)	0/ 20
Icterids:	
Great tailed grackle*	3 / 0
Brown-headed cowbird*	0/24
Brewer's blackbird*	0/ 5
Red-winged blackbird*	26/ 4
Bullock's oriole	0/ 2
Game birds:	
California quail	9/ 2

Group and species	Nº of birds sampled in CR/NWR
Finches:	
House finch *	3/ 5
Lawrence's goldfinch	2/ 0
Purple finch	1/ 0
American goldfinch	0/ 6
Shrikes and vireos:	
Warbling vireo	1/ 1
Buntings:	
Lazuli bunting	0/ 1
Tyrant flycatchers:	
Black phoebe*	9/ 5
Pacific slope flycatcher	1/ 15
Ash-throated flycatcher	1/ 7
Willow flycatcher	1/ 2
Western kingbird	0/ 1
Swallows:*	
Northern rough-winged swallow	3/ 0
Tree swallow	0/ 3

Group and species	Nº of birds sampled in CR/NWR
Wading birds:	
Green heron	1/ 0
Woodpeckers:	
Downy woodpecker	1/ 0
Northern flicker*	0/ 2
Nuttall's woodpecker	0/ 3
Wood warblers:	
Orange crowned warbler	1/ 4
Common yellowthroat	2/ 1
Townsend's warbler	0/ 1
Yellow-rumped warbler	18/ 29
Wilson's warbler	0/ 1
Yellow warbler	0/ 1
Pigeons and doves:	
Mourning dove*	1/ 2
Grosbeaks:	
Black-headed grosbeak	0/ 10
Thrushes:	
Hermit thrush	0/ 21

Group and species	Nº of birds sampled in CR/NWR
Wrentits:	
Wrentit	0/ 6
Hawks:	
Sharp-shinned hawk	0/ 1
Kinglets:	
Ruby-crowned kinglet	1/ 23
Wrens:	
Bewick's wren	0/ 4
House wren	0/ 2
Mimids:	
California thrasher	1/ 0
Northern mockingbird	0/ 13
Chickadees and their allies:	
Oak titmouse	2/ 1
White-breasted nuthatch	0/ 1
Bushtit	0/ 10
Corvids:	
California scrub-jay	0/ 4
Kingfishers:	
Belted kingfisher	1/ 0

*Bird species observed by the authors on produce fields of the study site and neighbor counties

Supplemental Information Table 2. Common name and scientific name of the bird species captured and sampled

Common name	Scientific name
White-crowned sparrow	*Zonotrichia leucophrys*
Song sparrow	*Melospiza melodia*
Lincoln's sparrow	*Melospiza lincolnii*
California towhee	*Melozon ecrissalis*
Golden-crowned sparrow	*Zonotrichia atricapilla*
Lark sparrow	*Chondestes grammacus*
Fox sparrow	*Passerella iliaca*
Spotted towhee	*Pipiloma culatus*
Savannah sparrow	*Passerculus sandwichensis*
Dark-eyed junco	*Junco hyemalis*
Great tailed grackle	*Quiscalus mexicanus*
Brown-headed cowbird	*Molothrus ater*
Brewer's blackbird	*Euphagus cyanocephalus*
Red-winged blackbird	*Agelaius phoeniceus*
Bullock's oriole	*Icterus bullockii*
California quail	*Callipepla californica*
House finch	*Haemorhous mexicanus*
Lawrence's goldfinch	*Spinus lawrencei*
Purple finch	*Haemorhous purpureus*
American goldfinch	*Spinus tristis*
Warbling vireo	*Vireo gilvus*
Lazuli bunting	*Passerina amoena*
Black phoebe	*Sayornis nigricans*
Pacific slope flycatcher	*Empidonax difficilis*
Ash-throated flycatcher	*Myiarchus cinerascens*
Willow flycatcher	*Empidonax traillii*
Western kingbird	*Tyrannus verticalis*
Northern rough-winged swallow	*Stelgidopteryx serripennis*
Tree swallow	*Tachycineta bicolor*
Green heron	*Butorides virescens*
Downy woodpecker	*Picoides pubescens*
Northern flicker	*Colaptes auratus*
Nuttall's woodpecker	*Picoides nuttallii*
Orange crowned warbler	*Oreothlypi scelata*
Common yellowthroat	*Geothlypi strichas*
Townsend's warbler	*Setophaga townsendi*
Yellow-rumped warbler	*Setophaga coronata*
Wilson's warbler	*Cardellina pusilla*
Yellow warbler	*Setophaga petechia*
Mourning dove	*Zenaida macroura*
Black-headed grosbeak	*Pheucticus melanocephalus*
Hermit thrush	*Catharus guttatus*
Belted kingfisher	*Megaceryle alcyon*
Wrentit	*Chamaea fasciata*
Sharp-shinned hawk	*Accipiter striatus*
Ruby-crowned kinglet	*Regulus calendula*
Bewick's wren	*Thryomanes bewickii*
House wren	*Troglodytes aedon*
California thrasher	*Toxostoma redivivum*
Northern mockingbird	*Mimus polyglottos*
Oak titmouse	*Baeolophus inornatus*
White-breasted nuthatch	*Sitta carolinensis*
Bushtit	*Psaltriparus minimus*
California scrub-jay	*Aphelocoma californica*

Supplemental Information on Materials and Methods

Specific methods for bird mist-netting and trapping

In the Commercial Ranch (CR), mist-nets were set approximately one hour before sundown close to a reservoir. They remained spun closed overnight and were opened by sunrise the following day, provided the temperature was higher than 4.4ºC (40ºF). Nets were checked every 20 minutes until nets were closed at noon or when the temperature rose to 32.2ºC (90º F). Nets were closed if the wind exceeded 6 mph sustained or if wind gusts exceeded 15 mph.

The same guidelines were followed at the National Wildlife Refuge (NWR). Nets were located at specific sites near water. As a bird banding and monitoring program has been in place on the NWR for almost 20 years, those sites were previously known for high bird activity and mist-netting success.

On both sites, a Tomahawk funnel ground trap was set the day before the sampling event. In addition, on the NWR a modified Australian crow trap was set by the cattle paddock to target Blackbirds and Brown-headed Cowbirds. On the CR we set up a crow trap to target American Crows specifically, as they were numerous on the ranch. This trap was baited with peanuts, fried corn chips, watermelon and others. The use of meat scraps or rotten eggs, which is usually advised to trap crows, was not possible because of the short distance to a produce field (<10m). The other ground traps were pre-baited with water and commercial songbird feed for feeders generally the day before the sampling event took place, and baited again the day of sampling.

Traps were activated after mist-nets were opened. Whereas the efficacy of the ground traps at the NWR was remarkable at the beginning, it largely dropped when California ground squirrels learned how to get in and out to steal the bait. Thus, we stopped using traps at this location by June 2016. For unknown reasons, the efficacy of ground traps in the CR was null.

Our capture success varied from 0.23 to 1.12 birds/net-hour and from 0 to 3.25 birds/trap-hour (being those traps that were ineffective not included in this calculation).

Details on the collection of samples

As birds were individually identified with aluminum bands provided by the USGS Bird Banding Laboratory, when we recaptured a bird we knew if it had been previously sampled by us. We re-sampled birds when at least a month had passed from the last sampling event. 15 and 16 birds were re-sampled at the CR and NWR respectively. Since they represent a small proportion of the total number of birds sampled, we report them as independent samples for simplification.

Those birds that were too large or too strong to be kept in paper bags were placed in a clean pillow case. After the release of the bird, we recovered the fecal material from the pillow case with a swab.
